# New Castle (Ind.) Medical Society

**Published:** 1857-12

**Authors:** Vindex Acestor

**Affiliations:** A Member


### NEW CASTLE (IND.) MEDICAL SOCIETY.

This Society met, Nov. 9th, President Beck in the chair.

Dr. John Rea reported several cases of phlegmasia dolens, wherein the good effects of the local application of the saturate alcoholic tinct. of iodine and bandaging the part were remarkable.

Dr. Isaac Mendenhall read a very interesting essay.

This is a model Society, and I am tempted to send you one or two Articles of its Constitution, for the inspection of those wishing to form new societies:

Art. XV. It shall be the duty of each member of this Society to keep a faithful record of each case of disease which he treats; noting the age, sex and condition of the patient, the cause where obvious, the type, symptoms, treatment, duration and termination of the disease; and, where practicable, the post-mortem appearances. The material facts of which record he shall embody in an intelligible form, and present it to the Society at its stated meeting in April (second Monday) of each year. And the Society shall have access to the case books of the members whenever called for.

Art. XVI. Each member shall also keep a faithful record of his obstetrical practice; embracing all the facts that are calculated to afford light upon the numerous difficult problems connected with the function of reproduction; a copy of which he shall present annually at the above stated meeting.

Art. XVII. The first stated meeting shall be the second Monday in April, for the purpose of embodying the reports of the cases of disease treated by the members during the preceding year, and this subject shall be pursued without delay until the said report is completed and adopted, a fair copy of which shall be preserved from year to year. The second stated meeting shall be the second Monday in June, and the third stated meeting shall be the second Monday in November.

At our last meeting, Nov. 9, the following officers were elected for the ensuing year.

President—John C. Beck, M. D., of Cadiz.

Secretary and Treasurer—John Rea, M. D., of New Castle.

Censors—Samuel Ferris, M. D., New Castle, Isaac Mendenhall, M. D., Ashland.

Dr. John Rea was appointed to read an essay at the next stated meeting (second Monday April).

The Society then adjourned to meet the 19th of January next, to hear the valedictory address of the former President.

VINDEX ACESTOR, M. D.,

A MEMBER.

